# Daclatasvir and Sofosbuvir Therapy Enhance Monocyte Phenotypic Changes in Naive Chronic Hepatitis C Patients: A Prospective Cohort Study

**DOI:** 10.1155/2019/9469567

**Published:** 2019-02-03

**Authors:** Hanan M. Fayed, Ali A. Ghweil, Mona M. AbdelMeguid

**Affiliations:** ^1^Clinical and Chemical Pathology Department, Qena Faculty of Medicine, South Valley University, Qena, Egypt; ^2^Hepatology, Gastroenterology and Tropical Medicine Department, Qena Faculty of Medicine, South Valley University, Qena, Egypt; ^3^Clinical Pathology Department, Faculty of Medicine for Boys, Al-Azhar University, Assiut, Egypt

## Abstract

**Background:**

Liver inflammation influences monocyte function, recruitment, and consequently inflammatory and fibrogenic responses. We aimed to investigate changes in the circulating monocyte phenotypes in response to Daclatasvir-Sofosbuvir (SOF/DCV) therapy in chronic hepatitis C (CHC) and relate findings to the viral kinetics and the fibrosis score.

**Methods:**

A longitudinal study involving 100 treatment-naïve patients and 30 healthy controls, tested for liver function, fibrosis scores (AST to platelet ratio index, FIB-4), and blood monocyte subsets based on CD14/CD16 expression by flow cytometer.

**Results:**

CHC patients had significantly lower albumin, higher ALT, AST, alkaline phosphatase, and increased fibrosis scores [Fib-4 (1.85±0.98) and AST to platelet ratio index (APRI) (0.6±0.35)], higher monocyte and eosinophil counts and lowered neutrophil to monocyte ratio (NMR), and lymphocyte to monocyte ratio (LMR) compared to week 12 and control. CHC patients had significantly increased median [classical (52.2% versus 25.8%,* P*=0.004) and inflammatory CD16^+^ monocytes (23.1% versus 13.58%,* P*=0.035)]. Therapy results in achievement of sustained virological response in 92% of cases, liver function improvement, and normalization of the inflammatory monocytes subsets. Monocyte counts showed positive correlation with viral load, calculated fibrosis scores (APRI and FIB-4 score), AST, ALT, ANC, and inverse correlations with serum albumin, leukocyte, eosinophil, NMR, and LMR. Multivariate regression found eosinophil count as predictors of CD16+ monocyte count in CHC patients.

**Conclusion:**

CHC infection promotes a proinflammatory and profibrotic monocytes profile. SOF/DCV therapy efficiently decreases viral load, reduces fibrosis potentials, attenuates monocyte activation, normalizes monocytes phenotypic abnormalities, and modulates monocyte subsets recruitment and differentiation later in the liver.

## 1. Introduction

Hepatitis C virus (HCV), a major public health problem in Egypt, is a primary cause of chronic liver disease, cirrhosis, and hepatocellular carcinoma [1].

Monocytes set up a 5-10% of peripheral leukocytes; they originate from bone marrow precursors and circulate in the peripheral blood for a few days before tissue migration where they display plasticity and provide a functionally diverse subset of macrophages, dendritic cells (DCs), fibrocytes, and fibroblasts [2]. And the monocytes recruitment is essential for effective control and clearance of infections [3], and the infiltrating monocytes act as phagocytes and antigen-presenting cells that offer a variety of proinflammatory, profibrotic cytokines, chemokines, and growth factors to repair damaged tissues [4].

There is a debate about the weak cellular immunity in patients with chronic HCV infection (CHC); this could be due to defective DC function, leading to weakness or absent or inappropriate T cell response to HCV [5], as HCV core protein induces monocytes IL-10/TNF-*α* secretion that triggers DC apoptosis and, secondarily, lowers IFN-*α* secretion [6]. In addition, monocytes play an important role in launching the adaptive immune response and operating the Th1/Th2 polarization by producing excessive inflammatory and immune-modulatory cytokines, such IL-10 and IL-12 that may weaken the antigen-presenting cells' ability to activate naive T cells and thus aid HCV replication and establish persistent infection [7]. Furthermore, monocytes trigger CD4^+^ T response and promote IL-17 producing T cell activities that further initiated chronic inflammatory and/or autoimmune diseases [8]. Moreover, monocytes also exert an antiviral function either directly (via the production of TNF-*α* and possibly other monokines) and indirectly (via IL-18-mediated stimulation of natural killer (NK) cells). However, patients with CHC infection have a reduced monocyte function and attenuated NK cell IFN*γ*-mediated responses [9], consequently the removal of the negative modulatory effects of NK cells on monocyte/macrophage function leading to their massive activation [10].

Human monocytes have three subsets based on the differential expression of lipopolysaccharide (LPS) receptor and immunoglobulin (Fc*γ*-III) receptor (CD14 and CD16) [11]. The majority of the population is CD14^high^ classical monocytes (CD14^++^CD16^−^) (~90%) and the minority population (~10%) includes the intermediate (CD14^++^CD16^+^) and the nonclassical CD14low (CD14+CD16++) monocytes [both collected as CD16+ monocytes]. Monocytes were anticipated to leave the bone marrow as classical cells, which can either directly attack inflamed tissues or differentiate into DC and/or macrophages, or they can differentiate into intermediate monocytes in the circulation [12]. The inflammatory nonclassical monocytes patrol along blood vessels to be involved in tissue homeostasis and regeneration [13], where they display features of tissue macrophages and perform inflammatory functions including phagocytosis, assembly of reactive oxygen species, nitric oxide, TNF-*α*, IFN-*γ*, and IL-6 [14]. Moreover, CD16+ monocytes are superior in CD4+ T cell activation, suggesting that they are more active inducers of inflammation. In addition, CD16+ monocytes effectively infiltrate the liver and then differentiate into macrophages, causing liver damage [15].

The alterations in the relative proportions of monocytes phenotype may have an association with disease progression that may permit the development of truthful biomarkers for disease severity or therapeutic strategies [16].

The use of direct-acting antivirals (DAAs) promotes great advances in the treatment of hepatitis HCV infection results in the achievement of a sustained viral response (SVR) after 12 weeks of treatment in more than 90% of CHC patients [17].

## 2. The Aim of the Work

The aim of this study was to characterize the circulating monocytes phenotype and to explore the effect of Daclatasvir and Sofosbuvir (SOF/ DCV) therapy on the monocytes subsets frequency and viral kinetics and relate findings with calculated fibrosis score in CHC patients.

### 2.1. Patients and Methods

It was a prospective case-control study, including 100 treatment-naïve chronic hepatitis C (CHC) patients attending the outpatient clinics of the Tropical Medicine & Gastroenterology Department, Qena University Hospital, from January 2017 to December 2017. Thirty age and sex-matched healthy subjects were selected as a control group. All eligible patients were included according to inclusion criteria approved by the national committee for control of viral hepatitis (NCCVH): age 18-75 years, HCV RNA positivity.

Exclusion criteria included coinfection with HBV or HIV, decompensated liver cirrhosis, inadequately controlled diabetes mellitus (HbA1c > 9%), hepatocellular carcinoma or extra-hepatic malignancy, previous IFN therapy, steroid intake or immune-suppressive therapy in the previous 6 months, drug addiction or alcohol abuse, and pregnancy.

Patients were assessed for HCV RNA at week zero (baseline), end of treatment, and 12 weeks after the end of treatment (SVR12). Patients were subjected to history taking, clinical examination, and routine laboratory work-up. All patients were treated with single oral daily fixed-dose combination therapy of Daclatasvir (60 mg) and Sofosbuvir (400 mg) (SOF/DCV) for 12 weeks.

Viral HCV RNA load by quantitative polymerase chain reaction assay (Cobas Amplicor, Cobas Taqman version 2.0) (Roche Diagnostics, Basel, Switzerland) HCV Roche, the lower detection limit 15 IU/ml), 12 weeks after the end of treatment was defined as SVR12, which is the main indicator of successful treatment.

### 2.2. Blood Samples

Ten ml venous blood samples were obtained by clean venipuncture [collected once from healthy controls and twice from CHC patients before therapy (w0) and (w12) after the end of treatment]; blood collected into two plain tubes and two K3 EDTA BD vacutainer tubes, one for CBC and second kept at 4°C to use for flow cytometry within 4 hours, and 3 plain tubes and after clotting and the serum was separated by centrifugation at 2000 RPM for 10 min.

All study subjects were subjected to the following investigations:

1. Complete blood count (CBC) using Cell DYN Emerald hematology analyzer (Abbott diagnostics, USA). The absolute cell count, percentages, and inflammatory indices that reflect the balance between host inflammation and immune response status as (lymphocyte to monocyte ratio (LMR); neutrophil to monocyte ratio (NMR)).
2. Laboratory routine liver biochemistry (ALT and AST levels, total bilirubin, albumin, total protein, and alkaline phosphatase); and fasting glucose (Cobas C311-Roche Diagnostics, Mannheim, Germany).
3. Calculation of liver fibrosis serum markers (Fibrosis score/indices):
  - AST to ALT Ratio (AAR) = AST/ALT. An AST/ALT ratio > 1 was found to be associated with advanced fibrosis [18].
  - Fibrosis-4 index (FIB-4) = Age [years] x AST [IU/L] ÷ (platelets [10^9^] x ALT [IU/L]). A FIB-4 index ≥ 2.67 had an 80% positive predictive value, and a value ≤ 1.30 had a 90% negative predictive value to diagnose advanced fibrosis [19].
  - AST to Platelet Ratio Index (APRI): AST value of 40 was used as the upper limit of normal (ULN). APRI was calculated with the following formula: APRI= AST/ULN AST X 100 ÷ platelet count. APRI showed high sensitivity and specificity and a significant correlation with both the stage of liver fibrosis and the grade of activity [20].
4. ** Phenotypic analysis of peripheral blood monocyte:** all antibodies were from (Beckman Coulter Immunotech-Marseille, France); using anti-human monoclonal antibodies (mAbs) anti-CD16 Fluoroisothio-cyanate (FITC) conjugated, anti-CD14 phycoerythrin (PE) conjugated and Mouse MoAb conjugated with FITC and PE Isotype-identical were used as negative controls.

For direct immunofluorescence labeling, 100 *μ*l of whole blood was added to each FCM tube and 10 *μ*L of each antigen-specific fluorochrome-conjugated MoAb or matched isotype controls, then samples were vortexed and incubated in dark for 15 minutes at room temperature. Following incubation, red blood cells lysing solution 2 ml (BD Biosciences) were added and incubated for 10 min at room temperature. Cells were centrifuged at 400xg for 5 min. The supernatant discarded and the cell pellet was washed twice with phosphate buffered saline (PBS) and then they were suspended in BD Biosciences fluid sheath for analysis.

All signals were acquired as the fraction of labeled cells within a cell gate set for 20,000 events at FACSCalibur flow cytometer (FCM) Becton Dickinson (BD Biosciences, San Jose, CA, USA); and Cell Quest software (version 4.0.2) was used to acquire and analyze the data.

### 2.3. Monocyte Gating

**Figure 1:**
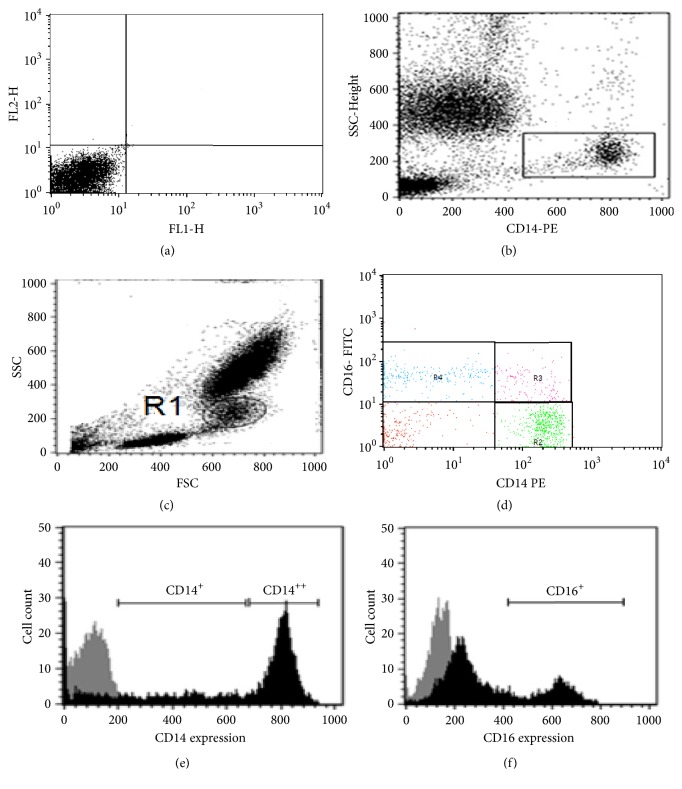
Flow-cytometer analysis of peripheral blood monocyte subsets: (a) isotopic control; (b) monocytes were assessed using an SSC/CD14 dot plot; (c) monocytes (R1) back-gated within the FCS/SSC dot plot; (d) the monocyte subsets within the (R1) population were assessed according to the expression of CD14/CD16; the three monocyte subsets were defined as classical CD14++CD16+ (R2), intermediate CD14+CD16+ (R3), and nonclassical CD14+/-CD16++ (R4) and small negative population for CD14-/CD16-, likely representing dendritic cells; (e) median fluorescence intensities (MFI) of CD14 and CD16; (f) MFI of CD16.

To ensure that only cells representing monocytes were analyzed, a positive gating strategy was employed to gate cells expressing CD14 at G1 using the linear side scatter (SSC) profiles and CD14 dot plot and back-gated G2 within the monocyte populations were analyzed defined by their size and granularity in the linear forward scatter (FSC) and side scatter (SSC) plots. Then the major monocyte subsets within the G2 population were analyzed based on the differential surface expression of CD14 and CD16 [21]. The three monocyte subsets were identified on a logarithmic scale and gates were then placed around the classical (CD14^++^CD16^−^), intermediate (CD14^++^CD16^+^), and nonclassical monocytes (CD14^+^CD16^++^) [Figure 1]; note that the (CD14^−^CD16^−^), i.e., lack both monocyte markers contains DCs. Cell surface expression was quantified as median fluorescence intensity minus the respective isotype control (MFI-MFI isotype), [Figure 1].

Absolute counts of monocyte subsets were calculated by multiplying the percentage of each subpopulation within the blood monocyte gate by the number of monocytes/*μ*L blood, as determined by CBC counts.

NB. For each monocyte subset the MFI of selected phenotypic markers were normalized by subtracting the MFI of the corresponding fluorescence-minus-one control channel.

### 2.4. Ethical Approval

The study protocol was approved by the institutional ethics committee and was conducted in compliance with the provisions of the Declaration of Helsinki, Good Clinical Practice guidelines, and local regulatory requirements and an informed consent obtained from all contributors.

### 2.5. Statistical Methods

All data were analyzed by a Statistical Package for Social Sciences (SPSS) software program (version 25) (IBM SPSS statistics, Inc., Chicago, IL, USA). As the data were not normally distributed, the nonparametric Kruskal-Wallis test was used for comparison of more than two independent samples and Pearson's correlation was used to assess relationships between variables. Linear regression analysis was used to determine predictor variables of the absolute monocyte count among the studied patients. All analyses were two-tailed and* P* < 0.05 was considered significant.

## 3. Results

### 3.1. Demographic and Clinical Characteristics of the Study Populations

The patients were all treatment-naïve CHC patients, 60 male/40 females, with a mean age of (52.2 ± 13.8 years), and (58%) had higher baseline plasma HCV RNA levels > 800 000 IU/ml. The regimen was well tolerated and 92% of CHC cases achieved sustained virologic response after 12 weeks of treatment (SVR12). The control group was 18 male/12 females with a mean age of 49 ± 10.5 years, Table 1.

### 3.2. Blood Chemistry of Study Groups

CHC patients (pretreatment) had significantly lower albumin, total protein, but had a significantly higher ALT, AST, alkaline phosphatase, and a significant increase in the calculated fibrosis scores Fib-4 and APRI but insignificant difference for AST/ALT ratio. The SOF/DCV treatment improves liver function significantly, resulting in a reduction of ALT, AST, alkaline phosphatase and fibrosis indices, and increase in albumin, Table 2.

### 3.3. CBC Analysis of Study Groups

CHC patients before treatment had significantly higher monocyte and eosinophil counts than posttreatment and had significantly lower NMR, LMR compared to after treatment and control group, Table 3.

### 3.4. Analysis of Monocyte Subsets

Monocyte subsets (classical, intermediate, and nonclassical) were significantly increased in CHC patients (w0) (before treatment) with significant higher classical monocytes count (52.2% versus 25.8%,* P* = 0.004), CD16^+^ monocytes (23.1% versus 13.58%,* P* = 0.035). SOF/DCV treatment results in significant reduction in the inflammatory monocyte subsets to a normal level. However, the classical monocytes remain higher than control, Table 4.

## 4. Correlations

In the CHC, the absolute monocyte count (basically inflammatory CD16+ monocytes) showed a significant positive correlation with the viral load and the calculated fibrosis scores (APRI and FIB-4 score), AST, ALT, ANC. Moreover, there were significant inverse correlations with parameters indicating the hepatic biosynthetic capacity (serum albumin), NMR, and LMR but there was no correlation with AST/ALT ratio. However; the absolute monocyte count showed a significant positive correlation with the total leukocyte count, absolute eosinophil count, alkaline phosphatase, Table 5.

In the CHC, the viral loads showed a significant positive correlation with the inflammatory monocytes (intermediate and nonclassical), Table 6.

The linear regression analysis performed to detect predictor variables of monocyte count, all the studied variables in the Univariate linear regression model significantly associated with an increase in the monocyte count (*P*-value < 0.05), Table 7.

The final model of the multiple regression model identified that LMR (posttherapy) and WBC as predictor variables of monocyte count (p < 0.05), Table 8.

Linear regression analysis of predictor variables of absolute CD16+ monocytes count and the Univariate linear regression model identified only eosinophil as a predictor variable of the absolute CD16+ monocytes (regression coefficient = 0.12,* P*-value = 0.043), Table 9.

## 5. Discussion

Macrophages are a heterogeneous cell population with different roles in the liver, including; phagocytosis, preserving immune tolerance and both promotion and resolution of inflammation and fibrosis [22]. Moreover, they were involved in the liver regeneration following injury [23].

The liver Kupffer cells (local resident self-renewing macrophages) from almost 80% of all body macrophages [24], which is constantly replenished by blood monocytes and is enhanced by the number of infiltrating monocytes in acute or chronic liver injuries yet can promote fibrogenesis by activating the profibrotic cytokine TGF-*β* [25]. Moreover, Liu and coworkers [26] confirmed that the reduction of circulating monocyte number and function were strongly associated with activation of systemic anti-inflammatory responses.

It is recognized that HCV mainly replicates in hepatocytes; however, monocytes with their phagocytic potential were found to contain the greatest HCV RNA loads [27]. In CHC patients, during the viremia, HCV circulates in the bloodstream with monocyte and the viral life cycle only goes to completion after a monocyte differentiation into macrophages in the confined hepatic environment [28].

The current study, CHC patients had elevated liver enzymes and decreased serum albumin that improved with therapy compared to controls.

CHC patient's had increased numbers of circulating eosinophils that were significantly reduced after SOF/DCV therapy; this was in agreement with Tarantino and colleagues [29] who established that hepatic infiltration by eosinophils was more frequent in older patients with a liver injury caused by steatosis and fibrosis. This could be as an effect of activated Kupffer cells, resulting in cytotoxicity to eosinophils with later degranulation, and release of mediators that enhances more inflammation, promoting cellular activation, generation of cytokines, and stimulation of the cyclooxygenase pathway that was toxic to various cell types that increases liver injuries [30]. Moreover, eosinophils have been related to the initiation and preservation of Th2 immune responses [31].

The CHC patients exhibit increased numbers of the circulating monocytes, with significantly increased all monocyte subsets (classical, intermediate, and nonclassical). And the expanded CD16+ monocytes were closely associated with liver injury as indicated by ALT, AST levels, and this was linked with chronic liver inflammation and fibrogenesis; these findings were in agreement with [15, 32, 33]. But in partial agreement with the finding of Ning and coworkers [34], who reported a lower frequency of classic monocytes in CHC patients that gradually increased to healthy control levels at (w12), the relatively higher classic monocytes count in our study at the baseline is a sign of the probable best therapeutic outcome in our patients.

Stansfield and Ingram [35] reported a consecutive expansion of the intermediate monocytes with the subsequent increase in the nonclassical monocytes suggesting that the intermediate monocytes can develop into the nonclassical monocytes. Besides Liaskou and colleagues [32] reported the development of the intermediate monocytes from the classical monocytes due to the effect of IL10 and IL4. However, our study did not detect these changes in the monocyte subsets; rather we found a reduction of the intermediate and the nonclassical monocyte subsets following therapy.

The CHC patient's CD16^+^ inflammatory monocyte was higher at w0 but decreased to normal levels following therapy at w12, and whether this is due to increased efflux from the blood into the inflamed liver or due to increased apoptosis in the circulation is unknown. This was in agreement with the finding of Ning and coworkers [34]. However, Liaskou and colleagues [32] reported that they could not detect any differences in monocyte counts or subsets in different etiologies of chronic liver disease, and they suggested that the changes in the monocyte compartment (either quantitative or qualitative) represent a constant reaction during liver disease progression and fibrogenesis.

In the CHC patient (either pretreatment or posttreatment), the absolute monocyte count showed significant positive correlation with total leukocyte count, ANC. This was in line with Zhang and colleagues [15] who found that the proinflammatory CD16+ monocytes and neutrophils accumulated in the liver, leading to inflammatory responses and liver damage in chronic hepatitis B patients.

The current study, serum HCV viral load, was negatively correlated with the hepatic biosynthetic capacity (serum albumin), NMR, LMR, but positively correlated with the serum ALT and AST levels, the serological fibrosis markers (APRI and FIB-4), the absolute monocytes count, the inflammatory CD16+ monocytes, and MFI 14. It appeared that the monocyte maturation and/or activation are related to the active viral replication rather than the virus itself. This was in agreement with Zimmermann and colleagues [33] and they suggested that the quantitative and qualitative changes in the monocyte compartment represent the disease progression, inflammation, and fibrogenesis. But partially consistent with Zhang and colleagues [15] who found that the CD16+ subset frequencies in peripheral blood were positively correlated with serum ALT levels and negatively correlated with HCV viremia.

In the present study, the AMC and inflammatory CD16+ monocytes were positively correlated with fibrosis indices of APRI and FIB-4. Few studies have confirmed the high sensitivity, specificity of the APRI fibrosis index, and they found a significant correlation between APRI with both the stage of liver fibrosis and the grade of activity [36]. Moreover, the APRI and FIB-4 had a high negative predictive value, so can be used for determination patients with mild fibrosis and for the discrimination and differentiation of mild to moderate fibrosis from severe fibrosis [37].

We found that SOF/DCV therapy significantly reduces the number of proinflammatory monocytes. This may have resulted from suppressed activation and maturation of monocytes, downregulation of CD16, and/or decreased turnover and trafficking of this subset of myeloid cells from the bone marrow.

In the current study, in CHC patient (either pretreatment or posttreatment), the absolute DCs count (CD14^−^CD16^−^) was significantly lower compared to controls; this could be explained by the fact that DCs apoptosis was a common mechanism of virus immune evasion during HCV infection [8].

The logistic regression analysis established eosinophil count as a predictor of CD16+ monocyte count in CHC patients.

*Limitations of This Study.* The small number, the single group, open-label design, treatment-naïve cases, and therefore the clinical efficacy of viral load monitoring during the short-duration DAA therapy need to be further evaluated through larger studies, and in particular, in patients with cirrhosis and/or past treatment experience. Phenotypic analysis of peripheral blood monocytes has not been performed on liver mononuclear cells and the restriction of permitted DAAs regimens. Finally, the functional status of circulating monocytes was not evaluated.

**In conclusion,** CHC infection promotes proinflammatory and profibrotic monocytes. SOF/DCV therapy improves patient's immunity to clear the HCV virus as indicated by the normalization of altered circulating monocyte phenotypes, the decrease of inflammatory monocytes, reduced fibrosis potentials, and the reduced viral replication. The changes in peripheral immune cells may be an early predictor and a potential marker for the prognosis and (SOF/DCV) therapy outcome. Thus, the peripheral blood monocytes phenotypic analysis is useful for the evaluation of the liver functional status as it is correlated with the degree of liver inflammation and fibrosis.

## Figures and Tables

**Table 1:** The baseline clinical and laboratory data of study populations.

	**CHC patient**
Age (years) Mean ±SD	52.2±13.8

Male/female	60/40

**HCV RNA viral load (IU/ml)**	

Mean ±SD, log_10_ IU/ml	6.09±0.55

Median (range) log_10_ IU/ml	6.09 (5.18-6.91)

≥ 800,000 IU/ml	58%

< 800,000 IU/ml	42%

**Table 2:** Comparison of laboratory investigation findings in CHC patients and healthy controls.

**Lab. parameters**	**Before**	**After**	**Control**	**P value**
**Total bilirubin (mg/dl)**				0.144994
Mean ±SD	0.77±0.37	0.59±0.30	0.72±0.71	
Median (range)	0.7(0.3-1.7)	0.5(0.2-1.4)	0.45(0.25-1.3)	

**Albumin (g/dl) **				**< 0.00001** **∗**
Mean ±SD	3.82±0.31	4.33±0.44	4.46±0.42	
Median (range)	4(3.1- 4.5)	4(4-5)	4.4(4-5)	

**Total protein (g/dl)**				0.156156
Mean ±SD	7.51±0.56	7.86±0.71	7.28±0.99	
Median (range)	7.6(6.6-8.5)	7.9(6.2-9.8)	7.4(6.3-7.9)	

**ALT (IU/L)**				**0.00001** **∗**
Mean ±SD	46.23±25.82	18.56±10.59	21±5.66	
Median (range)	34(14-94)	15.5(6-56)	19(4-37)	

**AST (IU/L)**				**0.00001** **∗**
Mean ±SD	47.46±21.0	23.58± 12.88	24±4.24	
Median (range)	37.5(21-90)	20.5(4-60)	24(8-37)	

**Alkaline phosphatase (IU/L)**				**0.0108** **∗**
Mean ±SD	190.42±42.59	181.96±41.12	127.6±12.02	
Median (range)	179.5(134-300)	173(125-280)	128(111-145)	

**F. glucose (mg/dl)**				0.57441
Mean ±SD	124.92±62.18	132.33±65.68	109±30.41	
Median (range)	89(59-317)	114(60-369)	30.41(77-165)	

**AST/ALT ratio**				0.169847
Mean ±SD	1.49±0.44	1.40±0.77	0.97±0.16	
Median (range)	1.01(0.65-2.57)	1.36 (0.29-4.5)	0.99(0.69-1.22)	

**Fib-4 score**				**0.007** **∗**
Mean ±SD	1.85±0.98	1.38±0.77	0.65±.30	
Median (range)	1.59(0.58-4.12)	1.21(0.32-3.67)	0.58(0.32-1.07)	

**APRI**				**0.0003** **∗**
Mean ±SD	0.6±0.35	0.29±0.19	0.29±0.18	
Median (range)	0.46(0.18-1.3)	0.23(0.04-0.88)	0.26(0.16-0.51)	

*∗*Significant; APRI: AST to platelet ratio index; fibrosis-4 index.

**Table 3:** Comparison of CBC findings in chronic HCV patients and healthy controls.

	**Before**	**After**	**Control**	**P value**
**Hb (g/dl)**				0.223
Mean ±SD	14.11±1.25	13.50±1.35	13.68±0.42	
Median (range)	14.3(11.8-17.2)	13.55(10.8-16.5)	13.9(11.2-15.6)	

**Platelet count (10** ^**9**^ **/l)**				0.516
Mean ±SD	223.350±72.390	219.770±54.770	222.000±32.520	
Median (range)	201(133-479)	215(122-323)	218(172-271)	

**WBC (10** ^**9**^ **/l)**				0.311
Mean ±SD	6.722±2.754	6,315±1,872	6.920±1.414	
Median (range)	6.010(3.000-14.600)	5.850(3.600-10.800)	7.200(4.000-8.800)	

**ANC (10** ^**9**^ **/l) **				0.695
Mean ±SD	3,766±2.425	3,378±1.273	3.964±1.273	
Median (range)	3,091(1.200-11.826)	3,470(0.966-5.785)	4,320(2400-559)	

%** Neutrophil **				0.789
Mean ±SD	52.69±13.03	52.96±12.28	56.8±12.02	
Median (range)	53.5 (26-81)	55(23-73)	60(43-65)	

**ALC(10** ^**9**^ **/l)**				0.614
Mean ±SD	2.286±677	2.403±960	2.530±1.230	
Median (range)	2.258 (1.050-4.214)	2.297(0.980-5.724)	2.448(1.440-3.344)	

%** Lymphocyte **				0.817
Mean ±SD	37.31±11.94	38.35±9.70	37.4±12.02	
Median (range)	36(11-65)	37.5(20-67)	36(26-53)	

**AMC(10** ^**9**^ **/l)**				**0.003** **∗**
Mean ±SD	497±0.275	286±145	313± 14	
Median (range)	400 (132-1287)	278(41-651)	264(160-602)	

%** monocyte **				**0.00001** **∗**
Mean ±SD	7.58±2.39	4.58±1.65	4.4±0.71	
Median (range)	7.5(3-11)	4.5(1-8)	4(3-7)	

**AEC (10** ^**9**^ **/l)**				**0.024** **∗**
Mean ±SD	20.19±15.65	12.72±8.32	12.96±1.41	
Median (range)	17.4(0-68.4)	12.4(0-27.6)	8(6-26.4)	

%** Eosinophil **				0.076
Mean ±SD	3.19±2.76	2.039±1.34	1.8±0.71	
Median (range)	2(1-12)	2(1-5)	2(1-3)	

**NMR**	8.27±4.5	14.82±11.17	13.86±0.47	**0.022** **∗**
	7.01(3.5-20)	12(5.2-60)	14.33(9.29-18.67)	

**LMR**	5.75±3.17	10.58±7.19	9.97±6.13	**0.008** **∗**
	5.3(1.6-16.25)	8.15(3.3-37)	9(3.71-17.67)	

*∗*Significant; AEC: absolute eosinophil count; ALC, absolute lymphocyte count; AMC, absolute monocyte count; ANC, absolute neutrophil count; LMR: lymphocyte to monocyte ratio. NMR: neutrophil to monocyte ratio.

**Table 4:** Comparison of monocyte subsets in CHC patients and healthy controls.

	**Pre-treatment**	**Post-treatment**	**Control**	**P value**
**Classical (CD14++ CD16-) **%				**0.025** **∗**
Mean ±SD	50.05±18.86	47.57±28.17	25.4±8.84	
Median (range)	52.2(1.82-78)	58.5(0.26-82)	25.8(12.6-41)	

**Classical (CD14++ CD16-) x10** ^**9**^ **/l**				**0.0062** **∗**
Mean ±SD	244±171	151±117	58±13	
Median (range)	193(7-704)	152(70-379)	51(27-94)	

**Intermediate(CD14++CD16+) **%				**0.0001** **∗**
Mean ±SD	5.86±3.99	2.20±2.23	1.39±3.49	
Median (range)	5.3(0.11-15)	1.7(0-8)	0.37(0.13-5.3)	

**Intermediate(CD14++CD16+) x10** ^**9**^ **/l**				**0.00003** **∗**
Mean ±SD	28±24	6±7	2±6	
Median (range)	22(0.4-114)	4(0-22)	1(0-9)	

**Non-classical (CD14+CD16++) **%				**0.0005** **∗**
Mean ±SD	17.98±7.16	7.96±6.64	11.33±0.92	
Median (range)	16.6(0.12-55)	6.89(0.01-24.6)	11.35(4-19)	

**Non-classical (CD14+CD16++) x10** ^**9**^ **/l**				**0.008** **∗**
Mean ±SD	104±138	22±21	28±0.2	
Median (range)	69(0.7-708)	19(0-76)	25(7-56)	

**CD16+ monocyte **%				**0.00002** **∗**
Mean ±SD	24.78±13.25	10.17±7.58	12.495±4.41	
Median (range)	23.1(0.23-58)	9.8(0.01-28.2)	13.38(4-19.85)	

**CD16+ monocyte x10** ^**9**^ **/l**				**0.0009** **∗**
Mean ±SD	132±134	29±24	30±6	
Median (range)	95(0.9-737)	25(0-92)	30(7-57)	

**MFI CD14 **				**0.0003** **∗**
Mean ±SD	29.59±7.06	24.44±9.83	13.66±1.1	
Median (range)	30.25(15.3-45)	25.5(2-45)	25.5(7.8-21.2)	

**MFI CD16 **				0.5315
Mean ±SD	492.43±252.10	438.23±282.43	371.33±2.83	
Median (range)	387(177-1087)	438(26-1137)	394(201-556)	

*∗*Significant; MFI: median fluorescent intensity.

**Table 5:** Correlation between viral load, absolute monocyte count, inflammatory monocyte, and other laboratory parameters.

**Laboratory parameters**	**HCV viral load**		**AMC**		**CD16+ monocyte**	
	**R**	**P value**	**r**	**P value**	**R**	**P value**
**Viral load **log_10_ IU/ml	-	-	**0.489**	**0.0002** **∗**	**0.6212**	**<0.0001** **∗**

**APRI**	**0.503**	**0.00015** **∗**	**0.32**	**0.0207** **∗**	**0.3903**	**0.00423** **∗**

**AST/ALT ratio**	-0.169	0.23229	0.1403	0.3212	0.1042	0.4622

**Fib-4 fibrosis score**	**0.345**	**0.0122** **∗**	**0.3157**	**0.023** **∗**	**0.3188**	**0.0213** **∗**

**Albumin (g/dl)**	**-0.530**	**<0.00001** **∗**	**-0.455**	**0.0007** **∗**	**-0.6114**	**<0.0001** **∗**

**Total protein (g/dl)**	**-0.331**	**0.01641** **∗**	-0.082	0.5607	-0.1142	0.4203

**ALT (IU/L)**	**0.559**	**<0.00001** **∗**	**0.325**	**0.019** **∗**	**0.4154**	**0.0022** **∗**

**AST (IU/L)**	**0.580**	**<0.00001** **∗**	**0.408**	**0.003** **∗**	**0.5278**	**<0.00001** **∗**

**Alkaline phosphatase (IU/L)**	0.067	0.63583	**0.323**	**0.019** **∗**	0.1967	0.16216

**Fasting glucose (mg/dl)**	-0.230	0.10062	-0.093	0.5099	-0.0238	0.8668

**Monocyte (10** ^**9**^ **/L)**	**0.489**	**0.00024** **∗**	-	-	**0.6755**	**<0.0001** **∗**

**Monocyte%**	**0.526**	**<0.00001** **∗**	-	-	**0.6907**	**<0.0001** **∗**

**Hb (g/dl)**	0.189	0.17936	-0.0758	0.5931	0.0345	0.80803

**Platelets count**	-0.107	0.45179	0.032	0.8211	0.0607	0.66898

**WBC (10** ^**9**^ **/L)**	0.086	0.54315	**0.5591**	**<0.00001** **∗**	0.2233	0.1115

**ANC (10** ^**9**^ **/L)**	-0.035	0.8074	0.4174	0.0021	0.0929	0.51248

**ALC (10** ^**9**^ **/L)**	0.078	0.58527	0.233	0.097	0.0592	0.6767

**# Eosinophil (10** ^**9**^ **/L)**	0.244	0.08177	**0.496**	**0.0002** **∗**	**0.3292**	**0.01715** **∗**

**NMR **	**-0.4501**	**0.00081** **∗**	**-0.540**	**<0.00001** **∗**	**-0.5664**	**<0.00001** **∗**

**LMR**	**-0.456**	**0.00079** **∗**	**-0.803**	**<0.0001** **∗**	**-0.6348**	**<0.00001** **∗**

*∗*Significant; ALC, absolute lymphocyte count; AMC, absolute monocyte count; ANC, absolute neutrophil count; LMR: lymphocyte to monocyte ratio. NMR: neutrophil to monocyte ratio.

**Table 6:** Correlation between viral load and other laboratory parameters.

	**HCV viral load**		**HCV viral load**		**HCV viral load**	
	**r**	**P value**	**r**	**P value**	**R**	**P value**
	**Events**		**Percentage**		**Absolute count**	

**(CD14++ CD16-) classical **	0.230628	0.09999	-0.10021	0.47969	**0.275768**	**0.05** **∗**

**(CD14++CD16+) intermediate**	**0.49564**	**0.00019** **∗**	**0.502554**	**0.00015** **∗**	**0.60335**	**<0.0001** **∗**

**(CD14+ CD16++) non-classical**	**0.5477**	**<0.00001** **∗**	**0.527427**	**<0.00001** **∗**	**0.612396**	**<0.0001** **∗**

**(CD16+) inflammatory monocyte **	**0.41676**	**0.00212** **∗**	**0.58368**	**<0.00001** **∗**	**0.62119**	**<0.0001** **∗**

**(CD14- CD16-) Dendritic cells**	0.18844	0.18095	-0.00622	0.9651	0.25414	0.0690

**MFI 16**	0.1428	0.31254				

**MFI 14**	**0.33246**	**0.01604** **∗**				

*∗*Significant.

**Table 7:** The univariate and multivariate linear regression of predictor variables of absolute monocyte count after treatment.

**Variables **	**Univariate regression analysis**		**Multivariate regression analysis**	
	**Regression coefficient (CI)**	**P value**	**Regression coefficient (CI)**	**P value**
**LMR post**	**- 12.34 (-19.05–-5.63)**	**0.001** **∗**	**- 14.16 (-32.06–3.74)**	0.114

**NMR post**	**- 7.13 (-11.69–-2.57)**	**0.004** **∗**	**2.69 (-8.49–13.89)**	0.62

**Eosinophil **	**0.97(0.35–1.59)**	**0.004** **∗**	**0.24 (- 0.31–0.78)**	0.378

**Lymph**	**0.08 (0.02–0.13)**	**0.007** **∗**	**0.02 (-0.11–0.16)**	0.718

**WBC**	**0.05 (0.03–0.08)**	**< 0.001** **∗**	**0.05 (-0.05–0.15)**	0.296

**Neutrophil **	**0.05 (0.007–0.09)**	**0.024** **∗**	**-0.04 (-0.047–0.06)**	0.418

*∗*Statistically significant.

**Table 8:** Final model of linear regression of predictor variables of absolute monocyte count after treatment.

**Variables **	**Regression coefficient (CI)**	**P value**
**LMR post**	**- 10.7 (-15.66–-5.76)**	**< 0.001** **∗**

**WBC**	**0.07 (0.03–0.097)**	**< 0.001** **∗**

**∗**Statistically significant.

**Table 9:** The univariate linear regression of predictor variables of absolute count of CD 16 + monocyte count after treatment.

**Variables **	**Regression coefficient (CI)**	**P value**
**LMR post**	**- 1.24 (-2.56– 0.09)**	0.066

**NMR post**	**- 0.84 (-1.69–0.007)**	0.052

**Eosinophil **	**0.12 (0.004–0.23)**	**0.043** **∗**

**Lymphocyte**	**0.01 (-0.006–0.015)**	0.379

**WBC**	**0.003 (-0.002–0.008)**	0.23

**Neutrophil **	**0.002 (-0.006–0.01)**	0.562

**∗**Statistically significant.

## Data Availability

The data used to support the findings of this study are available from the corresponding author upon request.
